# A Cross-Sectional Study of Alzheimer-Related Proteins in Women with Polycystic Ovary Syndrome

**DOI:** 10.3390/ijms25021158

**Published:** 2024-01-18

**Authors:** Alexandra E. Butler, Abu Saleh Md Moin, Thozhukat Sathyapalan, Stephen L. Atkin

**Affiliations:** 1 Research Department, Royal College of Surgeons of Ireland, Busaiteen P.O. Box 15503, Bahrain; amoin@rcsi.com (A.S.M.M.); satkin@rcsi.com (S.L.A.); 2 Academic Endocrinology, Diabetes and Metabolism, Hull York Medical School, Hull HU6 7RU, UK; thozhukat.sathyapalan@hyms.ac.uk

**Keywords:** polycystic ovary syndrome, amyloid-associated proteins, Alzheimer’s disease

## Abstract

Polycystic ovary syndrome (PCOS) is the most common endocrine condition in women of reproductive age, and several risk factors found in PCOS are associated with an increased risk of Alzheimer’s disease (AD). Proteins increased in AD have been reported to include fibronectin (FN) fragments 3 and 4 (FN1.3 and FN1.4, respectively) and ApoE. We hypothesized that Alzheimer-related proteins would be dysregulated in PCOS because of associated insulin resistance and obesity. In this comparative cross-sectional analysis, aptamer-based SomaScan proteomic analysis for the detection of plasma Alzheimer-related proteins was undertaken in a PCOS biobank of 143 women with PCOS and 97 control women. Amyloid precursor protein (APP) (*p* < 0.05) and amyloid P-component (APCS) (*p* < 0.001) were elevated in PCOS, while alpha-synuclein (SNCA) (*p* < 0.05) was reduced in PCOS. Associations with protective heat shock proteins (HSPs) showed that SNCA positively correlated with HSP90 (*p* < 0.0001) and HSP60 (*p* < 0.0001) in both the PCOS and control women. Correlations with markers of inflammation showed that APCS correlated with interleukin 6 (IL6) (*p* = 0.04), while Apolipoprotein (Apo) E3 correlated with TNF-alpha (*p* = 0.02). FN, FN1.3, FN1.4 and ApoE were all elevated significantly (*p* < 0.05). An AD-associated protein pattern with elevated FN, FN1.3, FN1.4 and ApoE was found in PCOS, in addition to elevated APP and reduced SNCA, which was the same as reported for type 2 diabetes (T2D) with, additionally, an elevation in APCS. With the AD biomarker pattern in PCOS being very similar to that in T2D, where there is an association between AD and T2D, this suggests that larger prospective cohort studies are needed in women with PCOS to determine if there is a causal association with AD.

## 1. Introduction

Polycystic ovary syndrome (PCOS) is the most common endocrine condition in women of reproductive age, characterized by menstrual irregularity, anovulatory infertility and hirsutism. There is also an increase in the prevalence of metabolic features, including type 2 diabetes (T2D); hypertension; and, potentially, cardiovascular disease [1,2] and fatty liver disease [3], the mechanism of which is still unclear, though insulin resistance and obesity-related inflammation have been implicated [1,4]. There are additional hereditable genetic factors [5,6] that contribute to and interact with epigenetic and environmental factors [7].

Several risk factors found in PCOS are associated with an increased risk of Alzheimer’s disease (AD). Predictions suggest the worldwide prevalence of patients with dementia, including those with AD, will increase to 115.4 million in 2050 [8]. AD accounts for up to 80% of all dementia cases [9], and there is evidence of an increased risk of T2D patients developing AD [10,11,12], which is important given that 10% of PCOS women may develop diabetes [13]. The pathophysiology of AD is not fully understood but includes inflammation [14] and protein misfolding [15]; β-amyloid is formed from native amyloid precursor protein (APP), and misassembly of amyloid precursor protein (APP) fragments results in toxic oligopeptide formation [14]; and β-amyloid fragments, mainly the amyloid beta 42 (Aβ-42) isoform, exhibit cytotoxic properties. Tau protein is involved in tubulin formation, and the formed and deposited neurofibrillary fibers are the result of the hyperphosphorylation of the Tau protein [14].

Women with PCOS are recognized as more commonly suffering from mood disorders, such as anxiety and depression, and sleep disturbances compared with women without PCOS [16]. Further, there is some evidence to suggest that there may be a link between Alzheimer’s disease and PCOS. Studies have reported that women with PCOS may be at increased risk of developing cognitive impairment and Alzheimer’s disease later in life [17]. This could be due to the underlying insulin resistance that is associated with PCOS, which can affect cognitive function and increase inflammation [18]. In a functional magnetic resonance (MRI) study, women with PCOS showed a decreased amplitude of low-frequency fluctuation, associated with poor executive performance and depressive disorders, and this negatively correlated with the plasma insulin level in subjects with insulin resistance [19]. Others have shown that reduced functional connectivity within the right frontal lobe upon MRI is related to the high luteinizing hormone (LH) levels present in PCOS [20]. Studies have suggested that the cognitive dysfunction in PCOS is, in part, due to hormonal dysregulation, including increased testosterone [21] and insulin [22].

A study utilizing aptamer-based proteomics in a comparison between AD, frontotemporal dementia (FTD) and controls showed that six proteins, fibronectin (FN), FN1.3, FN1.4, von Willebrand factor (VWF) and extracellular matrix protein 1 (ECM1), were discriminatory with elevation in AD in comparison with both FTD and controls [23].

**Figure 1:**
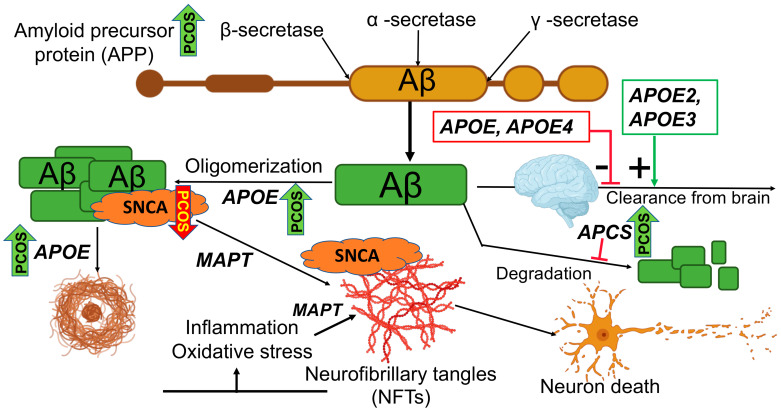
A schematic to illustrate the biology of amyloid-beta (Aβ)-induced neuronal death. The enzyme secretases act on amyloid-beta precursor protein (APP) to cleave the protein into three fragments. Sequential cleavage via β-secretases and γ-secretases produces the amyloid-beta (Aβ) peptide fragments. No Aβ is formed if the APP is cleaved by α-secretase. Aβ undergoes oligomerization with the help of Apolipoprotein E (ApoE). Aβ oligomers form senile (neuritic) plaque. Aβ oligomers, in association with ApoE and microtubule-associated protein tau (MAPT), form neurofibrillary tangles that eventually lead to neuron death. Aβ clearance from the brain is positively regulated by ApoE proteins (ApoE2, ApoE3) and negatively regulated by ApoE and ApoE4. Aβ degradation is also regulated by the serum amyloid P component (APCS). Upward green arrows indicate the Alzheimer-related proteins upregulated in PCOS (APP, APCS and ApoE); downward red arrows indicate the Alzheimer-related protein (SNCA) that is downregulated in PCOS.

We previously reported that an elevation in plasma amyloid precursor protein (APP), which is associated with AD, and decreased alpha-synuclein (SNCA) were found in patients with T2D [24]. The proteins available in the SomaScan panel included APP, which is associated with a decrease in established Alzheimer’s disease [25]; the soluble form of tau, which has shown specificity to AD [26]; the amyloid P component has shown to be reduced in the sera of patients with AD [27]; and SNCA, which is involved in the pathophysiology of Alzheimer’s disease [28,29]; one study showed elevated levels of SNCA in AD compared with heathy controls [30]; the overexpression of pappalysin (PAPPA) has been shown to play a role in Aβ peptide accumulation in Alzheimer’s disease [31]; Apolipoprotein (Apo) E levels have been investigated in AD [32], with some reporting an increase [33], some a decrease [34] and some no change [35]; serum amyloid A1 (SAA1) is an acute-phase protein that may play a housekeeping role in healthy tissue, but increased expression has been shown in the brain in Alzheimer’s disease [36]; nerve growth factor (NGF) imbalance occurs in patients affected by AD [37]; plasma glial fibrillary acidic protein (GFAP) may be associated with amyloid burden in AD [38]; noggin rescues age-related stem cell loss in an animal model of neurodegeneration, and we found it to be decreased in type 2 diabetes [24,39] (Figure 1).

Given that 10% of subjects with PCOS will develop T2D [13], it is important to know if the two conditions share risk factors for the development of AD, which, if recognized, may lead to preventative therapeutic strategies. We, therefore, hypothesized that such a pattern of AD-related protein changes seen in T2D may be reflected in PCOS patients given the insulin resistance and increased risk of developing T2D that these subjects have; therefore, we analyzed Alzheimer-related protein levels in women with and without PCOS from a UK Biobank and whether these proteins were correlated with markers that are common in the inflammatory pathway.

## 2. Results

Baseline data for the 137 PCOS subjects and 97 controls are shown in Table 1. The two cohorts were age-matched, but subjects with PCOS had a greater body mass index (BMI), increased insulin resistance, hyperandrogenemia and increased C-reactive protein (CRP, an inflammatory marker).

The results of the SomaScan analysis of Alzheimer-related proteins are shown in Table 2 for the PCOS and control women.

### 2.1. Levels of Alzheimer-Related Proteins in PCOS

**Figure 2:**
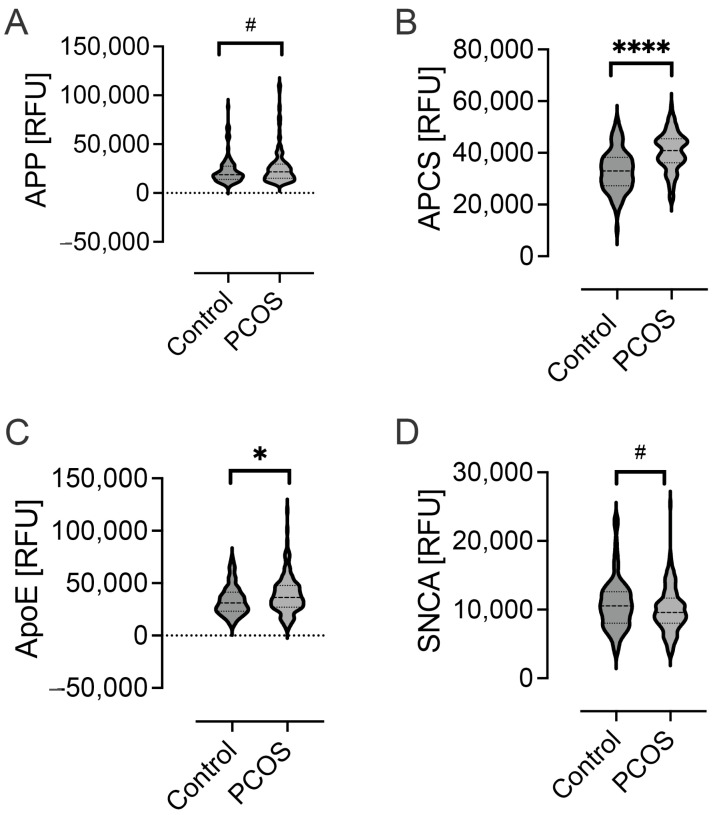
Alzheimer-related plasma protein levels in women with and without polycystic ovary syndrome (PCOS). APP (*p* < 0.05) (**A**); APCS (*p* < 0.001) (**B**); and ApoE (*p* < 0.01) (**C**) were elevated in PCOS, while SNCA (*p* < 0.05) (**D**) was reduced in PCOS. ^#^ *p* < 0.05; * *p* < 0.01; **** *p* < 0.0001.

Significantly elevated levels of APP (*p* < 0.05) and APCS (*p* < 0.001) were seen in PCOS, while SNCA (*p* < 0.05) was reduced in PCOS (Figure 2). The levels of other Alzheimer-related proteins, namely, PAPPA, MAPT, ApoE2, ApoE3, ApoE4, SAA, NOG and ApoA1, were comparable between PCOS subjects and controls (Table 2). A comparison between APP, APCS, SNCA and ApoE in T2D showed that the proteins APP and APCS in PCOS mirrored the same changes seen in a T2D cohort [24], and indeed, a comparison between the PCOS and T2D plasma values showed comparable levels, though SNCA was significantly higher in PCOS (*p* < 0.01)(Table 3). FN (*p* < 0.01), FN1.3 (*p* < 0.05), FN 1.4 (*p* < 0.01), ApoE (*p* < 0.01) and VWF (*p* < 0.04) were significantly elevated, while ECM1 did not differ (Table 2).

### 2.2. Stratification of Hyperandrogenemia, Obesity, Insulin Resistance, Glucose and Age in the PCOS Population

To determine if other facets inherent to PCOS may have given rise to these differences, stratification of the PCOS population based on non-hyperandrogenemia vs. hyperandrogenism (total testosterone ≤ 1.5 nmol/L vs. 1.6 nmol/L and above); non-obesity vs. obesity (BMI ≤ 30 kg/m^2^ vs. >30 kg/m^2^); non-insulin resistance vs. insulin resistance (HOMA ≤ 2 vs. >2); non-hyperglycemia vs. hyperglycemia (fasting plasma glucose ≤ 5.9 mmol/L or 6.0 mmol/L and higher); and older vs. younger women (upper tertile of age compared to lower tertile) was undertaken. There was a difference when stratifying according to hyperandrogenemia, with APCS, ApoE and ApoA1 higher in hyperandrogenemia (*p* < 0.01, *p* < 0.04 and *p* < 0.01, respectively), while PAPPA was decreased in hyperandrogenemia (*p* < 0.01). There was no difference seen when stratifying according to obesity, insulin resistance, fasting glucose or age.

### 2.3. Correlation Analyses

For the four proteins that differed between PCOS subjects and control women (APP, APCS, ApoE and SNCA), correlations with age; BMI; insulin resistance (HOMA-IR); testosterone; circulating levels of selected inflammatory proteins and protective heat shock proteins (HSPs); interleukin 6 (IL6); tumor necrosis factor alpha (TNFa); HSP90AA1 (termed heat shock protein 90; HSP90); and HSPD1 (termed heat shock protein 60; HSP60)) were performed.

**Figure 3:**
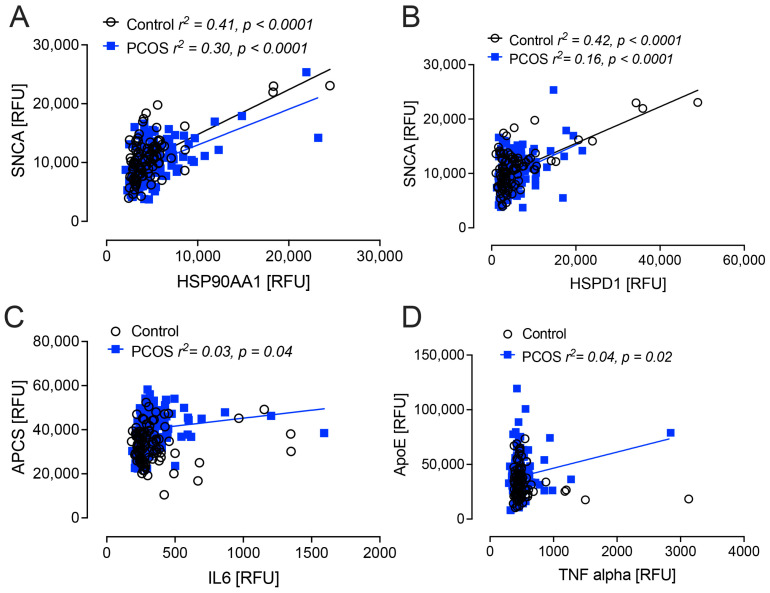
Correlations of Alzheimer-related proteins that differed between women with and without polycystic ovary syndrome (PCOS). SNCA correlated positively with HSP90AA1 (HSP90) (**A**) and HSPD1 (HSP60) (**B**) in both PCOS and control women (*p* < 0.0001). APCS correlated positively with IL6 (*p* = 0.04) (**C**), and ApoE correlated positively with TNFa (*p* = 0.02) (**D**) only in women with PCOS. Controls: black open circles; PCOS: blue squares.

SNCA correlated positively with HSP90AA1 and HSPD1 in both the PCOS and control women (*p* < 0.0001). APCS correlated positively with IL6 (*p* = 0.04), and ApoE correlated positively with TNFa (*p* = 0.02) only in women with PCOS (Figure 3). There was no association found for APP, APCS, ApoE, NGF, GFAP and SNCA with age, BMI, HOMA-IR or testosterone.

## 3. Discussion

Here, we report changes in Alzheimer-related plasma proteins in women with PCOS in comparison with women without PCOS, and intriguingly, the pattern of protein changes, with higher APP and lower SNCA, that was seen in this PCOS cohort was reflected in the basal samples of a cohort of subjects with T2D using the same aptamer-based technology, and those, in turn, reflected findings reported for AD [40,41,42]. In addition, the serum levels of APP and APCS were comparable between those with PCOS and T2D, though SNCA (decreased in both PCOS and T2D) was higher in PCOS versus T2D. β-amyloid is a product of APP that is also associated with a decrease in established Alzheimer’s disease [25]; however, it is not clear what plasma APP levels are found in those at risk of developing Alzheimer’s disease and whether an elevation may precede the subsequent decrease in established disease. APCS is also associated with a decrease in established Alzheimer’s disease [27], but its levels in those at risk and in pre-Alzheimer’s disease are not known. Studies have suggested the involvement of alpha-synuclein in the pathophysiology of Alzheimer’s disease [28,29], and one study showed elevated plasma levels for SNCA in Alzheimer’s disease compared with healthy controls [30], though others have not [43]. These findings are, therefore, important given a report showing AD-related proteins in T2D and the relationship of T2D with AD, and the findings here for the same AD-related proteins in PCOS are supported by an aptamer-based proteomic study that showed discriminatory differences in six proteins in AD (FN, FN1.3, FN1.4, VWF and ECM1) that were elevated in AD in comparison with both FTD and controls [23]; here, all but ECM1 were elevated in PCOS in comparison with controls.

When stratified according to demographic parameters, hyperandrogenemia showed increased APCS, ApoE and ApoA1, whereas PAPPA was decreased. The role of testosterone in Alzheimer’s disease has been reported in men where a lower testosterone level was associated with AD [44]. The role of testosterone in the development of AD in women is unclear, with a Mendelian randomization study suggesting that there was no association between testosterone and AD, though PCOS was not investigated in that study [45]; however, the results here suggest that further investigation is warranted, as the increase in APCS and the decrease in PAPPA related to testosterone may be protective [27,31].

It has been suggested that women with PCOS may have an increased risk of AD, with documented changes in cognition [17,19,20,21,22], and it is well recognized that patients with T2D have an increased risk of developing AD [10,11,12]. The question then arises as to whether women with PCOS have the same risk as those with T2D for developing AD. It is recognized that the mean and variability of insulin resistance seen in PCOS is of similar magnitude to that seen in T2D [46], and there is an increasing body of literature suggesting that the underlying insulin resistance associated with PCOS is responsible for alterations in cognitive function and, additionally, increases inflammation [18]. Insulin resistance leads to changes in insulin signaling through the PBK/AKT and MAPK pathways, leading to metabolic effects and cell homeostasis [47]; changes in insulin resistance may contribute to the sustained chronic inflammatory response in the AD brain that facilitates neurofibrillary tangle development and β-amyloid plaques [48]. Obesity is common in both PCOS and T2D, and obesity is also associated with an increased risk of AD [49]. This, then, presents a complex milieu, with insulin resistance leading to increased obesity because of compensatory hyperinsulinemia [50]; however, conversely, obesity, through mechanisms of chronic inflammation, adipokine activation and mitochondrial dysfunction [18], leads to insulin resistance, so a vicious cycle may result. The inflammation that is consequent upon insulin resistance/obesity may then be reflected in the development of cognitive impairment and progression to AD [51,52]. In this study, few correlations with inflammatory markers were seen, with only APCS correlating positively with IL6 and ApoE correlating positively with TNF-alpha only in women with PCOS, but no other correlations between inflammatory markers and APP and SNCA were found. Furthermore, there was no apparent association of AD-related proteins APP, APCS, ApoE, NGF, GFAP and SNCA with age, BMI, HOMA-IR or testosterone.

AD is characterized by pathological features of amyloid deposition in the brain [53,54,55], and there are abnormal interactions between amyloidogenic proteins and cellular machinery and membranes [56] that are considered to be diseases of protein misfolding [57,58,59] and may be associated with HSPs. Some evidence of this was seen in this study, where SNCA correlated positively with HSP90AA1 and HSPD1 in both the PCOS and control women, suggesting that a reduction in SNCA may be detrimental and induce the activation of the protective HSP response.

AD is characterized by the accumulation of β-amyloid (Aβ) proteins in the brain, with the generation of Aβ from amyloid precursor protein (APP) being the critical step in the development of AD [60], and elevated serum levels have been reported [41,42] in accord with this report. Amyloid P component (APCS) has been reported in plaques and neurofibrillary tangles in Alzheimer’s disease [27], and it may decrease the proteolysis of Aβ deposits, resulting in further plaque formation [61]; therefore, increased serum levels may be detrimental, in keeping with our findings here. Alpha-synuclein induces the fibrillization of MAPT (tau) and is also involved in Alzheimer’s disease-related brain pathology through its interactions with Aβ [28]. It is, therefore, apparent that there is a suggestion of an increase in AD risk with the protein profile seen in PCOS, mirroring that seen in T2D.

The treatment recommendations for PCOS suggest that lifestyle management should be the first step, and women with PCOS should lose 5–10% of their body weight and undertake regular physical exercise [62]. Such lifestyle management has been found to be successful [63] but is often difficult to sustain [64], and it is unknown whether AD-related risk factors are improved. Bariatric surgery has been shown to have a marked effect on PCOS with a reduction in BMI, insulin resistance and androgen levels and the return of regular menses, but no reports on AD-related risk factors are available [65]; however, the current evidence suggests that early and sustained lifestyle changes may have a long-term beneficial effect on cognition and AD-related risk and, at the very least, would not be harmful.

The limitations of this study include that it was a cross-sectional study, and the study numbers were small. As the study only included a white Caucasian population, it would need to be repeated to take into account ethnic differences. PCOS phenotype A, which expresses all three of the diagnostic criteria, is reported to be associated with a higher risk of adverse metabolic and cardiovascular outcomes compared with other phenotypes, and phenotype D is the least severe [66]; therefore, the expression of AD-related proteins needs to be clarified for individual PCOS phenotypes. A subsequent validation of the protein changes described with additional quantitative methods would also add value to the findings. Potentially, PCOS animal studies would provide further information, particularly as brain histology would accompany blood findings. AD is characterized by amyloid deposits whose major protein component is beta A4; beta A4 is a product of APP that is associated with a decrease in established Alzheimer’s disease [25]; however, sAPPα and -β and their variants were not available on the SomaScan panel to measure. In addition, the comparison of the protein levels between PCOS and T2D was limited, as the studies were not run on the same plates contemporaneously. Adjusting for BMI and insulin resistance is very difficult, as both are so highly correlated with PCOS that regression adjustment for either or both would remove the effects of PCOS; therefore, determining if a decrease in AD-related risk factors is dependent on obesity and/or insulin resistance would require a population of PCOS subjects that were nonobese and not insulin resistant.

## 4. Materials and Methods

In a cross-sectional analysis, plasma levels of Alzheimer-related proteins were measured in women with PCOS (n = 137) who were recruited presenting to the endocrine clinic of Hull Royal Infirmary, UK, and subjects without PCOS (n = 97) recruited via advert to a PCOS biobank (ISRCTN70196169: 2012–2017), as approved by the Newcastle & North Tyneside Ethics Committee (reference number 10/H0906/17 and date of approval 6 June 2014) [67] and in accordance with the 1964 Helsinki declaration and its later amendments or comparable ethical standards; all subjects gave written informed consent.

The women were all white Caucasians residing within 30 km of the endocrine clinic center. Inclusion criteria of PCOS: PCOS was diagnosed according to the Rotterdam consensus [68], based on two out of three of those criteria, namely, clinical and biochemical evidence of hyperandrogenism (Ferriman–Gallwey score >8, free androgen index >4 (local laboratory reference level), total testosterone > 1.5 nmol/L (local laboratory reference level)), oligomenorrhea or amenorrhoea and polycystic ovaries diagnosed with transvaginal ultrasound. PCOS exclusion criteria: Any significant medical history; currently taking medication of any kind (including oral contraceptive pills or over-the-counter medication). Confounding diagnoses such as nonclassical 21-hydroxylase deficiency were appropriately screened, as detailed previously [67]. Inclusion criteria for control subjects: Regular menses, normal physical examination, polycystic ovaries excluded by ultrasound, no concomitant medical conditions and not on any medications. Diabetes was excluded in all subjects with an oral glucose tolerance test. No subject had AD, and there was no family history of stroke or a family history of AD. The demographic data for the PCOS and control cohorts are shown in Table 1 [67].

Patients fasted overnight, and subsequently, blood samples were collected, as well as baseline weight and blood pressure. Height, weight, waist circumference and body mass index (BMI) were recorded according to WHO guidelines [69]. BMI information was defined as weight in kilograms and height in centimeters with the formula kg/m^2^, according to WHO guidelines. Blood was withdrawn and prepared via centrifugation at 3500× *g* for 15 min, aliquoted and stored at −80 °C. Analysis for sex hormone-binding globulin (SHBG); insulin (DPC Immulite 200 analyzer, Euro/DPC, Llanberis, UK); and plasma glucose (to calculate HOMA-IR) (Synchron LX20 analyzer, Beckman Coulter, High Wycombe, UK) was undertaken. Free androgen index (FAI) was derived from total testosterone divided by SHBG ×100. Insulin resistance (IR) was calculated using the homeostasis model assessment (HOMA-IR; (insulin u glucose)/22.5). Serum testosterone was quantified using isotope-dilution liquid chromatography–tandem mass spectrometry (LC-MS/MS) [70]. Total cholesterol, triglycerides and high-density lipoprotein cholesterol (HDL-C) levels were measured enzymatically using a Synchron LX20 analyzer (Beckman-Coulter, High Wycombe, UK). Low-density lipoprotein cholesterol (LDL-C) was calculated using the Friedewald equation. All hormone assays were performed with an Abbott Architect i4000 immunoassay analyzer (Abbott Diagnostics Division, Maidenhead, UK).

Alzheimer-related plasma proteins were measured using the Slow Off-Rate Modified Aptamer (Soma)-Scan platform [71]. Calibration was based on standards as previously described [72].

Quantification of proteins was undertaken using an aptamer-based methodology: the Slow Off-Rate Modified Aptamer (SOMAmer)-based protein array [73,74]. In short, plasma samples collected in EDTA were processed according to the following manufacturer-recommended method: (1) Equilibration of analyte/primer beads binding-SOMAmers (using a photocleavable linker, the synthetic SOMAmer labeled with a fluorophore was conjoined to a biotin moiety). (2) Analyte–SOMAmers complexes were immobilized on a streptavidin-substituted base. (3) Ultraviolet (UV) light was used to cleave and, therefore, release, the analyte–SOMAmer complexes into solution. (4) Using biotinylation, immobilization of the analyte–SOMAmer complexes on a streptavidin support was accomplished. (5) Analyte–SOMAmer complexes were eluted, and the released SOMAmers were utilized as analyte quantification surrogates. (6) Quantification was accomplished through hybridization to SOMAmer-complementary oligonucleotides. Raw intensities, hybridization and median and calibration signals were normalized and standardized [71,72].

Using version 3.1 of the SomaScan assay, the following proteins were targeted: amyloid precursor protein (APP), alpha-synuclein (SNCA), amyloid P-component (APCS), pappalysin (PAPPA), microtubule-associated protein tau (MAPT), apolipoprotein E, ApoE2, ApoE3, ApoE4, serum amyloid A (SAA), noggin (NOG), ApoA1, nerve growth factor (NGF) and glial fibrillary acidic protein (GFAP). In addition, AD-associated proteins FN, FN1.3, FN1.4, VWF, ApoE and VWF were measured, together with the levels of the following inflammatory proteins and protective heat shock proteins (HSPs): interleukin-6 (IL6), tumor necrosis factor-alpha (TNFa), HSP90AA1 (HSP90) and HSPD1 (HSP60).

### Statistics

Power was based on the APP and SNCA protein changes reported to be different in T2D [75] (nQuery version 9, Statsol, San Diego, CA, USA). APP: For an alpha of 0.05 with an effect size of 0.85, a total of 62 subjects (31 per arm) would be needed for 80% power: SNCA: For an alpha of 0.05 with an effect size of 0.80, a total of 68 subjects (34 per arm) would be needed based on 90% power if these proteins were to be significantly different in PCOS. Visual inspection of the data was undertaken followed by Student’s *t*-tests for normally distributed data and Mann–Whitney tests for non-normally distributed data as determined by the Kolmogorov–Smirnov test. All analyses were performed using GraphPad Prism version 9.4.1 (San Diego, CA, USA).

## 5. Conclusions

These data show that an AD-associated protein pattern with elevated FN, FN1.3, FN1.4 and ApoE was found in PCOS, in addition to elevated APP and reduced SNCA, which was the same as that reported for T2D with, additionally, an elevation in APCS. With the AD biomarker pattern in PCOS being very similar to that in T2D, where there is an association between AD and T2D, this suggests that larger prospective cohort studies are needed in women with PCOS to determine if there is a causal association with AD.

## Figures and Tables

**Table 1:** Demographics and baseline hormonal and metabolic parameters of the polycystic ovary syndrome (PCOS) subjects and controls. Data presented are median (IQR).

Baseline Demographics	PCOS (*n* = 137)	Controls (*n* = 97)	*p*-Value
	Median (IQR)	Median (IQR)	
Age (years)	27.9 (11.0)	28.5 (11.0)	0.09
BMI (kg/m^2^)	33.0 (9.9)	25.0 (5.7)	<0.001
Body weight (kg)	93.2 (33.3)	68.9 (20.9)	<0.001
Waist Circumference (cm)	101(21)	78(14.9)	<0.001
Insulin (IU/mL)	9.0 (8.0)	5.7 (4.1)	0.001
HOMA-IR	2.6 (2.4)	1.3 (1.1)	<0.005
CRP (mg/L)	3.1 (4.7)	1.0 (1.7)	0.001
SHBG (nmol/L)	21.0 (26.5)	53.5 (37.0)	0.001
Testosterone (nmol/L)	1.4 (0.9)	1.0 (0.4)	<0.001
FAI	4.5 (5.3)	2.2 (1.9)	<0.001
FSH (IU/L)	4.9 (2.8)	5.6 (3.6)	0.09
LH (IU/L)	6.1 (5.5)	4.3 (5.4)	0.009
TSH (mU/L)	1.9 (0.4)	1.8 (0.4)	0.09
AMH (pmol/L)	40 (42.7)	18.1 (24.8)	<0.001
Baseline Glucose (mmol/L)	4.7 (0.5)	4.5 (0.6)	0.01
2 Hour Glucose (mmol/L)	5.6 (1.8)	4.9 (1.3)	0.01

BMI—body mass index; HOMA-IR—homeostasis model of assessment—insulin resistance; CRP—C reactive protein; SHBG—sex hormone-binding globulin; FAI—free androgen index; AMH—anti-Mullarian hormone; FSH—follicle-stimulating hormone; LH—luteinizing hormone; TSH—thyroid stimulating hormone.

**Table 2:** Levels of Alzheimer-related proteins in women with polycystic ovary syndrome (PCOS) versus controls. Data presented are median (IQR) of relative fluorescent units (RFUs).

	Control (*n* = 97) Median (IQR)	PCOS (*n* = 137) Median (IQR)	*p*-Value
APP	18,763 (13,615)	21,698 (14,260)	0.04
SNCA	10,544 (4528)	9589 (3468)	0.04
APCS	32,981 (10,795)	40,889 (9122)	<0.0001
PAPPA	10,809 (6030)	11,597 (5196)	0.49
MAPT	140 (45)	157 (64)	0.34
ApoE	31,169 (17,676)	36,361 (20,187)	0.01
ApoE2	261,224 (55,647)	265,481 (53,411)	0.67
ApoE3	195,313 (71,683)	211,003 (74,881)	0.06
ApoE4	207,878 (67,027)	218,393 (67,602)	0.22
SAA	723 (1348)	690 (1244)	0.51
NOG	2534 (889)	2307 (802)	0.48
ApoA1	14,679 (3003)	14,562 (3837)	0.33
GFAP	562 (312)	564 (246)	0.14
NGF	418 (94)	418 (104)	0.56
vWF	12,319 (5195)	13,040 (9478)	0.04
FN	14,898 (7663)	17,380 (14,450)	0.01
FN1.3	3100 (947)	3168 (1558)	0.02
FN1.4	66,449 (14,318)	71,627 (25,002)	0.007
ECM1	20,403 (4377)	19,721 (5860)	0.99

Amyloid precursor protein (APP); alpha-synuclein (SNCA); amyloid P-component (APCS); pappalysin (PAPPA); microtubule-associated protein tau (MAPT); Apolipoprotein E (ApoE); ApoE2; ApoE3; ApoE4; serum amyloid A (SAA); noggin (NOG); ApoA1; glial fibrillary acidic protein (GFAP); nerve growth factor (NGF); von Willebrand factor (vWF); fibronectin (FN); fibronectin fragment 3 (FN1.3); fibronectin fragment 4 (FN1.4); extracellular matrix protein 1 (ECM1).

**Table 3:** A comparison of key Alzheimer’s disease-related protein levels in a cohort of patients with type 2 diabetes (T2D) [24] and the women reported here with polycystic ovary syndrome (PCOS) plus their respective controls. Data are presented as median (IQR). Protein levels are reported as RFUs (relative fluorescent units). There was no difference between T2D and PCOS for APP and APCS, but SNCA was significantly higher in PCOS (* *p* < 0.01).

	T2D (*n* = 23)	Controls (*n* = 23)	PCOS (*n* = 137)	Controls (*n* = 97)
APP	24,206 (38,065)	14,004 (14,521)	21,698 (14,260)	18,763 (13,615)
APCS	34,918 (6658)	34,700 (8503)	40,889 (9122)	32,981 (10,795)
SNCA	5519 * (1595)	6644 (2606)	9589 * (3468)	10,544 (4528)
ApoE	32,267 (19,636)	33,762 (20,514)	36,361 (20,187)	31,169 (17,676)
GFAP	328 * (45)	354 (88)	564 * (246)	562 (312)
NGF	399 (81)	435 (134)	418 (104)	418 (94)

Amyloid precursor protein (APP), alpha-synuclein (SNCA), amyloid P-component (APCS), Apolipoprotein E (ApoE), glial fibrillary acidic protein (GFAP), nerve growth factor (NGF).

## Data Availability

All the data for this study will be made available upon reasonable request to the corresponding authors.
